# Epidemiological Distribution of Major Ectoparasites Species of Small Ruminant in the Case of Chemical Control Campaign in Welkait District, Tigray Region, Ethiopia

**DOI:** 10.1155/2020/4175842

**Published:** 2020-03-16

**Authors:** Berhe Leul, Afera Berihun, Kebede Etsay

**Affiliations:** ^1^Livestock Research Core Process, Humera Agricultural Research Center, Tigray Agricultural Research Institute, Humera, Ethiopia; ^2^Faculty of Veterinary Medicine, Mekelle University, P.O. Box 231, Mekelle, Tigray, Ethiopia

## Abstract

A cross-sectional study was carried out from November 2016 to May 2017 to identify the major ectoparasites species and potential risk factors in Welkait district western part of Tigray region. A total of 102 sheep and 324 goats were physically examined and samples were taken for laboratory analysis. *Rhipicephalus evertsi evertsi* with a prevalence of 58 (56.86%), *Amblyomma gemma* 12 (11.76%), *Amblyomma variegatum* 27 (26.47%), *Boophilus decoloratus* 7 (6.86%), and *Hyalomma anatolicum excavatum* 1 (0.98%) in sheep and *R*. *evertsi evertsi* 108 (33.02%), *A*. *gemma* 8 (2.47%), *A*. *variegatum* 158 (48.77%), and *R*. *B*. *decoloratus* 19 (5.86%) in goats were the most important tick species identified. Statistically significant difference (*p* < 0.05) was obtained in the prevalence of *A*. *gemma* (*x*^2^ = 14.981; *p*=0.001) and *A*. *variegatum* (*x*^2^ = 15.696; *p*=0.001) between sheep and goats and *R*. *B*. *decloratus* (*x*^2^ = 8.137; *p*=0.017), *A*. *variegatum* (*x*^2^ = 90.159; *p*=0.00*p*=0.00), and *A*. *gamma* (*x*^2^ = 18.642; *p*=0.00) in goats and *A*. *variegatum* (*x*^2^ = 71.081; *p*=0.00) and *R*. *B*. *decloratus* (*x*^2^ = 28.980; *p*=0.001) in sheep by agroecology. *R*. *evertsi evertsi* (*x*^2^ = 13.400; *p*=0.001) and *A*. *variegatum* (*x*^2^ = 13.511; *p*=0.001) in goats and *R*. *B*. *decoloratus* (*x*^2^ = 71.892; *p*=0.001) and *A*. *gemma* (*x*^2^ = 6.414; *p* = 0.040) in sheep were found to have statistically significant association (*p* < 0.05) in the prevalence among different body condition categories in the present study. *R*. *evertsi evertsi* (*x*^2^ = 6.557; *p*=0.010) and *R*. *B*. *decoloratus* (*x*^2^ = 4.856; *p*=0.028) in goats and *R*. *evertsi evertsi* (*x*^2^ = 5.776; *p*=0.016) in sheep by sex group and *R*. *evertsi evertsi* (*x*^2^ = 40.556; *p*=0.001) and A. *variegatum* (*x*^2^ = 7.214; *p*=0.007) in goats by age group were also statistically associated (*p* < 0.05). Infestation rate of *R*. *evertsi evertsi* (*x*^2^ = 7.136; *p*=0.008), *R*. *B*. *decoloratus* (*x*^2^ = 9.621; *p*=0.002), and *A*. *variegatum* (*x*^2^ = 10.372; *p*=0.001) in goats between flock type had statistically significant association (*p* < 0.05) in this study. The current result revealed that *Linognathus stenopsis* was the second highest prevalent ectoparasite with an overall prevalence of 0.00% in sheep and 25.93% in goats. There was a statistically significant difference (*p* < 0.05) in the prevalence of *L*. *stenopsis* (*x*^2^ = 32.940; *p*=0.001) between the two species and in body condition category (*x*^2^ = 10.700; *p*=0.005) in goats in the present study. Moreover, *Ctenocephalides canis* and *Ctenocephalides felis* were the flea species found in the present report. Significant variation (*p* < 0.05) in *C*. *canis* prevalence among different agroecology (*x*^2^ = 10.264; *p*=0.006) in goats and between adult and young age (*x*^2^ = 5.052; *p*=0.025) in sheep and (*x*^2^ = 21.267; *p*=0.001) in goats was obtained in the present study. *Sarcoptes scapie var*. *caprea* with a prevalence of 0 (0.00%) in sheep and 4 (1.23%) in goats had no significant association (*p* > 0.05) in all the risk factors considered. The present result indicated that ectoparasites especially tick species were more prevalent in small ruminants and may affect the wellbeing and productivity of goats and sheep in the study district. Therefore, well-coordinated and urgent control intervention should be conducted.

## 1. Introduction

Data from the estimation of [1] indicate that Ethiopia is a home for about 56.71 million cattle, 29.2 million sheep, 29.3 million goats, 9.9 million equines, 1.2 million camel, and 56.9 million poultry and Tigray region possess 4.6 million cattle, 1.8 million sheep, 4.3 million goats, 0.8 million equines, 0.6 million camel, and 6.2 million poultry of the country. However, the economic gain from these animals remains insignificant to Ethiopia and Tigray region compared to their huge number of livestock poulation. Different causes are responsible for the decrease in the production and productivity of small ruminants in Ethiopia. Among the different factors that influence the production and productivity of small ruminants, diseases caused by ectoparasites are the most important [2]. Ectoparasite infestations have worldwide distribution and are recognized as a major hurdle in the health of small ruminants and hamper the production efficiency. Lice, flea, ticks, sheep kid, and mange are the most important ectoparasites that are associated with the negative impacts on the health of small ruminants [3]. Ectoparasites may exert direct and indirect effects on small ruminants. The indirect effect may be expressed by discomfort and annoyance and self-wounding by scratching while the direct impact may be due to feeding on different body tissues of the animals such as blood, skin, and hair. Moreover, ectoparasites could cause an intense irritation on the animal body; as a result of this condition, the infested animals rub or bite their skin in objects and damaged their skin and became low quality and rejected by tannery due scarification [4, 5, 6]. Ectoparasites also act as disease transmitters from healthy to diseased animals by transporting disease-causing pathogens such as protozoan, bacteria, virus, and rickettsia [7, 8].

As reported by Kidanu [9], the occurrence of skin defects due to ectoparasite damage especially cockle lesions causes a rapid increase in the past 10–15 years in Ethiopia and holds the highest place as a cause of skin downgrading and rejection in small ruminants. This condition has prohibited computation in the international markets to export semiprocessed and processed skin of small ruminants in the country [4, 10]. Reports from Mulugeta et al. and Rahmeto et al. [11, 12] indicate that 35% of sheep and 56% of goat skins could have been downgraded and rejected due to ectoparasite impact in Ethiopian tanneries. Tanneries in Ethiopia reported that only 10 to 15% skin of small ruminants provided to the industries have top-grade quality while the rest have the lowest quality and have been rejected [13].

Recent reports from Ethiopia clearly indicated that ectoparasites have negative impacts on the quality of small ruminants' skin. Report from Wukro Sheba tannery Tigray region by Hagos et al. [14] on the infestation status of cockle lesion in sheep pelts by *D*. *ovis* and *M*. *ovinus* and sarcoptic mange infested goat pelts indicated that 100% and 92.5%, respectively, infestation were obtained in case of sheep pelts whereas 100% occurrence of cockle lesion was obtained from goat pelts. To reduce the impact of ectoparasites on skin quality and improve the economic gain from the skin of small ruminants, the Ministry of Agriculture and Rural Development (MoARD) of Ethiopia designed ectoparasites control campaign program which is conducted in Amara, Afar, and Tigray regions in 2011 [10]. During the campaign program, a number of sheep and goats were treated by spraying and dipping majorly using organophosphates (diazinon 60%) and in fewer cases using ivermectin in Tigray region. Despite the ectoparasites control activity conducted, problem of ectoparasites of small ruminants is active and still complaints are raised in the study area from small ruminants' owners. Therefore, this study focused on the identification of the most important ectoparasites species and the potential risk factors associated with the occurrence of the disease.

## 2. Materials and Methods

### 2.1. Study Area

Welkait district is located in the Western Zone of Tigray region surrounded by Tselemti district in east, Tahtay Adiabo district in north, Asgede Tsimbla district in northeastern, Kafta Humera district in north and northwestern, and Tsegede district in south and southwestern. It has three agroecological zones which constitute 3% highland, 37% midland, and 60% lowlands areas. The district is located 437 km away from the central city of Tigray regional state and 1220 km far from Addis Ababa. The annual temperature and rainfall of the district are 17.5–25°C and 700–1800 mm, respectively. This area lies in the ranges 677–2755 meters above sea level, and the district has a total human population of 163,939 which consists of 83,129 men and 80,810 women. From the total population, urban inhabitant's number is 14,843 which accounts for 9.1% of the total population and an estimated total area of 3811.18 square kilometers. Tekeze River is located in the eastern part of the district and bound 4, 882 km. Zarema, Kaza, Ruwasa, and Kalema are the four big rivers with many tributaries in the district. The vegetation cover of the district is 86,180 hectare and 4,960 hectare areas are covered by incense tree distributed especially along the big river Tekeze and lowland kebeles of the district [15].

### 2.2. Study Animals and Sampling Method

Indigenous sheep and goats of different sex and age groups, body conditions, agroecology, species, and flock type kept under extensive management system were used for the study. Nine localities (peasant association (PA)) were randomly selected from the district. The number of representative sample animals was proportionally allocated to the selected peasant associations and village based on the number of sheep and goats, and simple random sampling technique was used to select the representative sample animals from their flock. The minimum sample size required was estimated using the formula described by Thrusfield [16] considering 95% confidence interval with 5% desired absolute precision and an expected prevalence of 50%. The sample size calculated was 384. Accordingly, 102 sheep and 324 goats were examined.

### 2.3. Ectoparasites Collection and Identification

Ear, limb, under the tail, neck, shoulder, breast, ribs, back, flank, and rump areas of both sides of sheep and goat body were thoroughly examined by close inspection and parting the hairs against their natural direction after proper restraining. Ticks were removed from the host skins while retaining their mouthparts for identification using thumb forceps. Coat brushing technique was applied to collect lice from host skin. From clinically positive animals, specimens of lice ticks and fleas were collected, preserved in individual properly labeled universal bottle containing 70% alcohol, and transported to Mekelle University parasitological laboratory, and samples were examined by stereomicroscope. Identification was done based on their morphological features given by Walker et al. [17] for ticks and Soulsby and Wall and Shearer [18, 19] for flea and lice. In case of mange mite, first the hair was clipped and skin scraping was taken using scalpel blade by scraping the edge of the affected area until blood oozed as described by Chauhan and Agerwal [20]. The material that scrapped falls on paper and was held and transferred to a clean universal bottle, and the preserved samples in 10% formalin were transported to the Welkait district veterinary clinic for analysis. In the veterinary clinic laboratory, a few drops of 10% potassium hydroxide were added to the sample and allowed to stand for 30 minutes. A drop of the sediment was transferred to clean slide and covered with coverslip, and mites' identification was made under a low-power microscope according to Taylor et al. [5] and Wall and Shearer [19].

### 2.4. Data Analysis

The collected data were entered into Microsoft Excel datasheets and analyzed using STATA 11 statistical software (STATA Corporation, College Station, TX). The prevalence of the ectoparasites species was calculated by dividing the proportion of animals found positive for each ectoparasites species by the total number of animals examined. The difference in the prevalence of ectoparasites species in different risk factors considered was analyzed using the Pearson chi-square (χ^2^) test [16] adopted from the study of Fikre et al. [21].

## 3. Result

According to the present findings, the major tick species identified were *R*. *evertsi evertsi* with the prevalence of 58 (56.86%), *A. gemma* 12 (11.76%), *A*. *variegatum* 27 (26.47%), *B*. *decoloratus* 7 (6.86%), and *H*. *A*. *excavatum* 1 (0.98%) in sheep and *R*. *evertsi evertsi* 108 (33.33%), *A*. *gemma* 8 (2.47%), *A*. *variegatum* 158 (48.77%), and *B*. *decoloratus* 19 (5.86%) infestation rate in goats. Statistically significant difference (*p* < 0.05) was obtained in the prevalence of *A*. *gemma* (*x*^2^ = 14.981; *p*=0.001) and *A*. *variegatum* (*x*^2^ = 15.696; *p*=0.001) between sheep and goats in the present study. The second important ectoparasite identified in the present study was *L*. *stenopsis* with the prevalence of 0 (0.00%) in sheep and 84 (25.92%) in goats. There was a statistically significant difference (*p* < 0.05) in the prevalence of *L*. *stenopsis* (*x*^2^ = 32.940; *p*=0.001) between the two species of small ruminates. *C*. *canis* with a prevalence of 7 (6.86%) in sheep and 60 (18.52%) in goats was found in this study. Statistically significant difference (*p* < 0.05) was obtained in the prevalence of *C*. *canis* (*x*^2^ = 7.952; *p*=0.005) between sheep and goats in the present study (Table 1).

The present result indicated that total ectoparasites prevalence of 97 (91.51%), 98 (82.35%), and 51 (51.52%) was found in highland, midland, and lowland in goats, respectively. Statistically significant (*p* < 0.05) difference was found in the prevalence of total ectoparasites (*x*^2^ = 49.047; *p*=0.00) in different agroecological zones of the study area in goats. *B*. *decoloratus* (*x*^2^ = 8.137; *p*=0.017), *A*. *variegatum* (*x*^2^ = 90.159; *p*=0.00), and *A*. *gemma* (*x*^2^ = 18.642; *p*=0.00) were significantly associated (*p* < 0.05) among different agroecological zones. *C*. *canis* prevalence of 28 (26.42%) in highland, 23 (19.33%) in midland, and 9 (9.09%) lowland was obtained in the present study. There was significantly high association (*p* < 0.05) in *C*. *canis* (*x*^2^ = 10.264; *p*=0.006) prevalence with the categories of agroecology considered. Furthermore, the infestation rate of 76 (61.79%) in good, 106 (80.30%) in medium, and 64 (92.75%) in poor body conditioned goats of total ectoparasites was also obtained in the present report. Statistically significant association (*p* < 0.05) in total ectoparasites (*x*^2^ = 25.522; *p*=0.00) prevalence with different body conditioned goats was evidenced. *R*. *evertsi evertsi* (*x*^2^ = 13,400; *p*=0.001), *A*. *variegatum* (*x*^2^ = 13.511; *p*=0.001), and *L*. *stenopsis* (*x*^2^ = 10.700; *p*=0.005) showed significantly different association (*p* < 0.05) among the different categories of body condition status in goats (Table 2).

Species level prevalence of ectoparasites in goats was also considered by sex, age, and flock type. From the tick species identified, *R*. *evertsi evertsi* with the prevalence of 27 (24.11%) in male and 81 (38.21%) in female and *R*. *B*. *decoloratus* with the prevalence of 11 (9.82%) in male and 8 (3.77%) in female were found. There was a statistically significant association (*p* < 0.05) with *R*. *evertsi evertsi* (*x*^2^ = 6.557; *p*=0.010) *and R*. *B*. *decoloratus* (*x*^2^ = 4.856; *p*=0.028) prevalence between the two sex groups of goats. *R*. *evertsi evertsi* with the prevalence of 83 (49.40%) and 25 (16.03%) in adult and young (*x*^2^ = 40.556; *p*=0.00) age group, respectively, *A*. *variegatum* with a prevalence of 94 (55.95%) in adult and 64 (41.03%) in young age (*x*^2^ = 7.214; *p*=0.007) and *C*. *canis* with the prevalence of 15 (8.93%) in adult and 45 (28.85%) in young age (*x*^2^ = 21.267; *p*=0.00) had a statistically significant (*p* < 0.05) association between the age groups in the present study. Total ectoparasites prevalence of 112 (68.71%) in single flock and 134 (83.23%) in mixed flock was recorded in the present study. Significant association (*p* < 0.05) occurred between the two groups (*x*^2^ = 9.340; *p*=0.002) in total ectoparasites prevalence. 43 (26.38%) in single and 65 (40.37) in mixed flock *R*. *evertsi evertsi* (*x*^2^ = 7.136; *p*=0.008), 3 (1.84%) in single and 16 (9.94%) in mixed flock *R*. *B*. *decoloratus* (*x*^2^ = 9.621; *p*=0.002), and 65 (39.88%) in single and 93 (57.76%) in mixed flock *A*. *variegatum* (*x*^2^ = 10.372; *p*=0.001), *p*=0.002) prevalence had statistically significant association (*p* < 0.05) in this study (Table 3).

A total ectoparasites prevalence of 18 (85.71%), 11 (100.00%), and 46 (65.71%) in highland, midland, and lowland in sheep was found in the present study. Statistically significant (*p* < 0.05) effect occurred among agroecological conditions (*x*^2^ = 7.759; *p*=0.021). *R*. *B*. *decoloratus* with the prevalence of 7 (33.33%) in highland, 0 (0.00%) in midland, and 0 (0.00%) in lowland (*x*^2^ = 28.980; *p*=0.001), *A*. *variegatum* with the prevalence of 17 (80.95) in highland, 9 (81.82%) in midland, and 1 (1.43%) in lowland (*x*2 = 71.081; *p*=0.001), and *A*. *gemma* with the prevalence of 0 (0.00%) in highland, 0 (0.00%) in midland, and 12 (17.39%) in lowland (*x*^2^ = 6.217; *p*=0.045) in sheep showed significant difference (*p* < 0.05) by agroecology in the present result. *R*. *B*. *decoloratus* with the prevalence of 2 (5.00%) in good, 4 (11.11%) medium, and 1 (4.00%) in poor body condition (*x*^2^ = 71.892; *p*=0.001) and *A*. *gemma* with the prevalence of 5 (12.20%) in good, 1 (2.78%) medium, and 6 (24.00%) in poor body condition (*x*^2^ = 6.414; *p*=0.040) in sheep had a statistically significant (*p* < 0.05) association (Table 4).

In the present study, *R*. *evertsi evertsi* with a prevalence of 25 (73.53) in male and 33 (48.53) in female was found. There was a significant variation (*p* < 0.05) in the prevalence of *R*. *evertsi evertsi* (*x*^2^ = 5.776; *p*=0.016) between male and female. *C*. *canis* infestation with the prevalence of 0 (0.00%) in adult and 7 (11.48%) in young age group (*x*^2^ = 5.052; *p*=0.025) had statistically significant (*p* < 0.05) association (Table 5).

## 4. Discussion

The genera of *Amblyomma*, *Boophilus*, *Rhipicephalus*, and *Hyalomma* and their five species were identified in sheep and goats in this study. Accordingly, *R*. *evertsi evertsi* with the prevalence of 58 (56.86%), *A*. *gemma* 12 (11.76%), A. *variegatum* 27 (26.47%), *R*. *B*. *decoloratus* 7 (6.86%), and *H*. *A*. *excavatum* 1 (0.98%) in sheep and *R*. *evertsi evertsi* 108 (33.02%), *A*. *gemma* 8 (2.47%), *A*. *variegatum* 158 (48.77%), and *R*. *B*. *decoloratu*s 19 (5.86%) in goats were the most important tick species identified. Similar genera of ticks were identified by different authors in Ethiopia. Accordingly, *Amblyomma* with a prevalence of 10.09%, *Boophilus* of 8.77%, and *Rhipicephalus* of 6.58% in sheep and *Amblyomma* with a prevalence of 10.26%, *Boophilus* of 6.69%, and *Rhipicephalus* of 5.77% in goats were reported by Shibeshi et al. [22]. Together with the genera level, *Amblyomma* with the prevalence of 11.8%, *Boophilus* 2.48%, and *Rhipicephalus* 5.59% in sheep and *Amblyomma* with the prevalence of 66.15%, *Boophilus* 3.08%, and *Rhipicephalus* 3.08% in goats were reported by Jemere et al. [23]. This study indicated that, with the exception of numerically higher finding of *Amblyomma* in goats of the later study, generally the prevalence of ticks of the present study was more higher compared to these findings. The reasons for differences in prevalence among the different studies may be associated with difference in the method of chemical application, geographical difference, difference in species of the study animals, season of study, grazing system, and animal management. *A*. *variegatum* ranked the second most prevalent tick species identified in sheep while it was the highest prevalent species of all ectoparasites in goats. Statistically significant difference (*p* < 0.05) was obtained in the prevalence of *A*. *variegatum* (*x*^2^ = 15.696; *p*=0.001) between sheep and goats in the present study. The finding of *A*. *variegatum* in both sheep and goats with greater frequency of occurrence in this study is in agreement with the report of Mulugeta et al. [11], Jemere et al. [23], and Jafer et al. [24] who reported *A*. *variegatum* is the second highest prevalent species of all ectoparasites detected in sheep while it was the highest prevalent species of all ectoparasites reported in goats.

Statistically significant difference (*p* < 0.05) was obtained in the prevalence of *R*.*B*. *decloratus* (*x*^2^ = 8.137; *p*=0.017), *A*. *variegatum* (*x*^2^ = 90.159; *p*=0.00), and *A*. *gemma* (*x*^2^ = 18.642; *p*=0.00) in goats and *A*. *variegatum* (*x*2 = 71.081; *p*=0.00) and *R*. *B*. *decloratus* (*x*2 = 28.980; *p*=0.001) in sheep by agroecology in the present study. The prevalence of A. *variegatum* was higher in midland and highland in both sheep and goats. The result of this study coincides with the report of Tesfay et al. [25] as expressed that *A*. *variegatum* has higher infestation rate in Africa and is the vector of heartwater disease with higher distribution in the highland area which has grazing pastures and lower distribution in area with highest forest coverage and thorny bush. *A*. *gemma* was only found in lowland goats and sheep in the present study. This is because the highland agroclimate has higher humidity and inauspicious condition for *A*. *gemma* to survive and only this tick species is found to be confined to lowland and semiarid areas as reported by Pegram et al. [26]. Morel [27] also stated that *A*. *gemma* has wide distribution in woodland, bush land, wooded, and grassland in arid and semiarid areas located at an altitude of 500–1750 meters above sea level with 350 to 750 mm annual rainfall. *R*.*B*. *decoloratus* was found to have higher distribution in highland and midland goats whereas it was found to have higher distribution in highland and with zero prevalence in midland and lowland sheep. This report is in agreement with the report of Pegram et al. [26] as indicated *R*.*B*. *decoloratus* has higher distribution in highland and semihighland climatic condition with more than 800 mm annual rainfall and has similar distribution to *A*. *variegatum*. *R*. *evertsi evertsi* (*x*2 = 13.400; *p*=0.001) and *A*. *variegatum* (*x*2 = 13.511; *p*=0.001) in goats and *R*. *B*. *decoloratus* (*x*2 = 71.892; *p*=0.001) and *A*. *gemma* (*x*2 = 6.414; *p*=0.040) in sheep were found to have statistically significant association (*p* < 0.05) in prevalence among different body condition categories in the present study. Similar to this report, prevalence difference of ticks among different body condition categories has been reported on small ruminants by Nateneal and Tesfaheywet [28] and Seid [29]. As general concept infestation of tick is higher in poor body condition than in good body condition, this is because higher infestation of ticks resulted in poor body conditioning in animals due to consumption of higher amount of blood and body fluid from the body of the animal as described by Kedir and Petros [30].

The prevalence of *R*. *evertsi evertsi* was higher in female than in male goats (*x*^2^ = 6.557; *p*=0.010) but was higher in male than in female in case of sheep (*x*^2^ = 5.776; *p*=0.016) whereas the prevalence of *R*. *B*. *decoloratus* (*x*^2^ = 4.856; *p*=0.028) was higher in male than in female in goats in the present study. The prevalence of these tick species' infestation between sex groups had statistically significant association (*p* < 0.05). The result of the present study disagrees with the report of Jemere et al. [23] in the prevalence of *R*. *evertsi evertsi* who recorded higher prevalence in male than in female goats and higher *R*.*B*. *decoloratus* in female than in male in Wolmera District of Oromiya Region, Central Ethiopia, but similar result was reported by Tesfaheywetm and Muluneh [31] in case of *R*. *evertsi evertsi* prevalence in sheep from Western Shoa Zone, Oromia regional state, Ethiopia. Sex difference in the prevalence of tick species indicated that female animals are more affected by non-sex-related diseases than males because the ability of female animals to resist an infection is disturbed and because of decrease in immunity during parturition and lactation [32]. Higher infestation found in male in this study might be due to the small number of male in the flock and frequent contact with infested sheep and goats during sexual meeting as described by Tewodros et al. [33]. The current study has shown statistically significant (*p* < 0.05) difference in the prevalence of *R*. *evertsi evert*si (*x*^2^ = 40.556; *p*=0.00) and *A*. *variegatum* (*x*^2^ = 7.214; *p*=0.007) in adult and young age group of goats. Higher prevalence of these tick species was found in adult age than in young age group of goats. The result of the present study is in line with the report of Jemere et al. [23] and Tesfaheywetm and Muluneh [31] who reported higher prevalence of *R*. *evertsi evertsi* and *A*. *variegatum* in adult goats than in young goats. Younger animals are less infested by tick than older animal because younger animals are separately kept and grazing around home by owners and have lower risk of tick infection compared to animals graze in pasture as reported by Tesfaheywetm and Muluneh [31] and Rasmi et al. [34]. *R*. *evertsi evertsi* (*x*^2^ = 7.138; *p*=0.008), *R*. *B*. *decoloratus* (*x*^2^ = 9.621; *p*=0.002), and *A*. *variegatum* (*x*^2^ = 10.372; *p*=0.001) in goats had statistically significant association (*p* < 0.05) between flock size in this study. The prevalence these ectoparasites was higher in large flock size than in small flock size because in large flock size there is an overcrowding and consistent contact between small ruminants for prolonged times which enhances ectoparasites spread from infected to uninfected animals according to the report of Mulugeta et al. [11] and Madeira et al. [35].

The current result revealed that *L*. *stenopsis* was the second most prevalent ectoparasite with an overall prevalence of 0 (0.00%) in sheep and 84 (25.92%) in goats. The prevalence of lice in this study was in line in case of sheep with report from southern range land with 0.00% and 1.55% prevalence in sheep and goats, respectively [36]. This result is in contrast with the findings of Tewodros et al. [33] who found the *Damalinia* species to be the main lice species with a prevalence of 33.69% and 26.12%, respectively, in sheep and goats, and *Linognathus* spp (23.8%) in sheep not identified in the current study, but *Linognathus* spp (21.62%) in goats was somewhat similar to the present study. Prevalence reported by Mulugeta et al. [11] in three selected agroecological sites of Tigray was 11.5% for *L*. *africanus* and 15.3% for Damalinia spp in sheep which were again not found in this study and 27.9% for *L*. *africanus* in goats which was almost similar to the finding of this study but different in species. An overall prevalence of lice (49.85%) and (82.35%) *D*. *ovis* in sheep and 0.00% prevalence in goats from controlled and uncontrolled areas, respectively, were recently found in Arsi by Hailegebriel et al. [37]. The ectoparasite species and prevalence obtained in the current study were different in species and lower than the prevalence of ectoparasites observed in Arsi in sheep, but higher in goats following governmental intervention. Another report by Zewdu et al. [2] from in and around Sekela, Amhara region, indicated that *L*. *ovillus* (14.2%) and *D*. *ovis* (8.9%) were predominant in sheep and the lower rate of *L*. *stenopsis* (17.7%) was recorded in goats compared to this study. The highest prevalence of lice reported by Hailu [38] which was *Linognathus spp* (75.5%) and *D*. *ovis* (67.1%) in sheep was different than that reported in the present report. Lower prevalence rates were also reported in the present result which was different than the report of Asnake et al. [39] who found 14.6% for *L*. *ovillus* and 36.1% for *D*. *ovis* in sheep. Along with the above findings, more similar report from Wolayta Sodo by Yacob et al. [40] indicated an overall prevalence of lice of 25.7% in sheep and 0.00% in goats Linognathus spp which is contrary to the present finding. This difference may be due to difference in agroecological and climatic conditions. Moreover, difference in production practices and chemical intervention, stress condition, feeding and housing conditions, and quarantine of newly introduced animals may also contribute to the fluctuation of lice infestation [22]. There was a statistically significant difference (*p* < 0.05) in the prevalence of *L*. *stenopsis* (*x*^2^ = 32.940; *p* = 0.001) between sheep and goats in the present study. In the present result, body condition of the goats was significantly associated (*p* < 0.05) with the prevalence of *L*. *stenopsis* (*x*^2^ = 10.700; *p* = 0.005) infestation. Higher prevalence was found in poor body condition than in medium and very good body conditions. Similar higher prevalence of Linognathus species in poor body conditioned goats than in good body conditioned goats was reported by Sisay et al. [41], but it disagrees with the report of Ethiopian Sheep and Goats Productivity Improvement Program (ESGPIP) [13] and Jemere et al. [23] who reported insignificant difference in the prevalence of *L*. *stenopsis* in poor and good body conditioned goats. Prevalence variation among differnt body conditions of goats may be due to differences in management and feeding : well-nourished animals have higher resistance to disease compared to poor-nourished animals [31].

*C*. *canis* with a prevalence of 7 (6.86%) in sheep and 60 (18.52%) in goats and *C*. *felis* infestation rate of 0 (0.00%) in sheep and 5 (1.4%) in goats were found in the present study. The present finding was not in agreement with the report of Tesfaye et al. [42] who reported prevalence of 45 (16.1%) in sheep and 14 (12.2%) in goats *C*. *felis* and 3 (1.1%) in sheep and 1 (0.9%) in goats *C*. *canis* at Bahir Dar Veterinary Clinic. On the other hand, (8.51%) *C*. *canis* and (4.8%) *C*. *felis* in sheep and (7.73%) *C*. *canis* and (11.3%) *C*. *felis* in goats reported by [43] from around Kombolcha was almost similar in the prevalence of *C*. *canis* and higher in the prevalence of *C*. *filis* in sheep but, lower in *C*. *canis* and higher prevalence *C*. *filis* in goats than the present study. This incongruity recorded in the prevalence of *Ctenocephalides* species among different authors may be due to differences in management and agroecological and climatic conditions. In the present study, there was a statistically significant difference (*p* < 0.05) in the prevalence of *C*. *canis* (*x*^2^ = 10.264; *p* = 0.006) in goats among different categories of agroclimatic zones. Higher prevalence was found in highland followed by midland and lowland, respectively. Prevalence of *Ctenocephalides* species is increased if the humidity is higher. Temperatures range of 21 to 30°C and 70% humidity are required by female flea to lay eggs [44, 45]. Significant variation (*p* < 0.05) in the prevalence of *C*. *canis* between adult and young age group of both sheep (*x*^2^ = 5.052; *p* = 0.025) and goats (*x*^2^ = 21.267; *p* = 0.00) was obtained in the present study. Higher prevalence of *C*. *canis* was obtained in young age group of sheep and goats than in adult age group. This result is inconsistent with the report of Yacob et al. [40] who reported insignificant difference in the prevalence of *C*. *canis* between adult and young age group of sheep and goats. Similar higher prevalence of *C*. *canis* in young age group than in adult was reported by Tesfaye et al. [42]. The higher prevalence in the younger animals might be associated with the shorter hair and thinner skin in young animal in which flea can easily access the skin and penetrate it without difficulty [42].

*S*. *scapie var*. *caprea* with a prevalence of 0 (0.00%) in sheep and 4 (1.23%) in goats was found in the present study. This result disagrees with the report of Mulugeta et al. [11] who reported 1.3% *Sarcoptes scabiei var. ovis* in sheep and 12.5% *S*. *scabiei var*. *caprae* in goats and with the report of Tesfaheywet and Misgana [46] who reported 2.5% *Sarcoptes scabiei var. ovis* in sheep and 5.43% *S*. *scabiei var*. *caprae* in goats. This result further disagrees with the report of Desalegn et al. [43] who reported 47.1% *Sarcoptes scabiei* from east Wollega zone, northwest Ethiopia. Lower prevalence in mange species obtained in this study than in other study areas might be due to the intensive control campaign conducted by the regional government for the past three to four years before the study time.

## 5. Conclusion

*R*. *evertsi evertsi*, *A*. *gemma*, *A*. *variegatum*, R. *B*. *decoloratus*, and H.A. excavatum were the important tick species identified in this study. *L*. *stenopsis* is the second important ectoparasite found followed by *C*. *canis*, *C*. *felis*, and *S*. *scapie var*. *caprea* in the present finding. Almost all of those ectoparasites species associated with all the risk factors considered. In view of the present study, it is possible to conclude that although ectoparasites control campaign was conducted in the study district, different ectoparasites species are still present and many sheep and goats are suffering from ectoparasites problem in the study area and tannery found in region complains about the quality of skin provided by skin and hid collectors of the districts. Lacks of awareness about the extent of the problems among owners, inaccessibility for control schemes, and poor efficiency of chemical control campaign have contributed to have different types of ectoparasites species in the area after the control campaign. Effective extension system and programs that could raise public knowledge on the effect of ectoparasites and further detailed study on seasonal variability and epidemiology of ectoparasites species as well as acaricidal test should be done in the study area.

## Figures and Tables

**Table 1:** Species and host-based ectoparasites prevalence.

External parasites	Species external parasites	Total (*n* = 426)	Sheep (*n* = 102)	Goats (*n* = 324)	*X* ^2^	*p* value
		Positive (%)	Positive (%)	Positive (%)		
Tick	*R*. *evertsi evertsi*	165 (38.96)	58 (56.86)	108 (33.33)	0.763	0.382
	*A*. *gemma*	20 (4.69)	12 (11.76)	8 (2.47)	14.981	0.001
	*A*. *variegatum*	185 (43.43)	27 (26.47)	158 (48.77)	15.696	0.001
	*R*. *B*. *decoloratus*	26 (6.10)	7 (6.86)	19 (5.86)	0.135	0.713
	*H. A. excavatum*	1 (0.23)	1 (0.98)	0 (0.00)		
Lice	*L*. *stenopsis*	84 (19.72)	0 (0.00)	84 (25.92)	32.940	0.001
Flea	*C*. *canis*	67 (15.73)	7 (6.86)	60 (18.52)	7.952	0.005
	*C*. *felis*	5 (1.17)	0 (0.00)	5 (1.54)	1.593	0.207
Mange	*S*. *scapie var*. *caprea*	4 (0.94)	0 (0.00)	4 (1.23)	1.260	0.262
Overall (grand total)		321 (75.35)	75 (73.53)	246 (75.93)		

H.A. excavatum = *Hyalomma anatolicum excavatum*.

**Table 2:** Ectoparasite species distribution in goats by agroecology and body condition scores.

Species of ectoparasites	Agroecology				Body condition scores			
	Highland (*N* = 106)	Midland (*N* = 119)	Lowland (*N* = 99)	χ^2^ (*p* value)	Good (*N* = 123)	Medium (*N* = 132)	Poor (*N* = 69)	χ^2^(*p* value)
*R*. *evertsi evertsi*	32 (30.19)	49 (41.18)	27 (27.27)	5.402 (0.067)	27 (39.13)	55 (41.67)	26 (21.14)	13.400 (0.001)
*R*. *B*. *decoloratus*	11 (10.38)	7 (5.88)	1 (1.01)	8.137 (0.017)	5 (4.07)	7 (5.30)	7 (10.14)	3.087 (0.214)
*A*. *variegatum*	68 (64.15)	81 (68.07)	9 (9.09)	90.159 (0.00)	44 (35.77)	76 (57.58)	38 (55.07)	13.511 (0.001)
*A*. *gemma*	0 (0.00)	0 (0.00)	8 (8.08)	18.642 (0.00)	4 (3.25)	4 (3.03)	0 (0.00)	2.233 (0.328)
*L*. *stenopsis*	36 (33.96)	25 (21.01)	23 (23.23)	5.437 (0.066)	20 (16.26)	39 (29.55)	25 (36.23)	10.700 (0.005)
*C*. *filis*	2 (1.89)	1 (0.84)	2 (2.02)	0.618 (0.734)	3 (2.44)	0 (0.00)	2 (2.90)	3.553 (0.169)
*C*. *canis*	28 (26.42)	23 (19.33)	9 (9.09)	10.264 (0.006)	23 (18.70)	22 (16.67)	15 (21.74)	0.777 (0.678)
*S*. *scapie var*. *caprea*	3 (2.34%)	1 (0.79%)	0 (0.00)	4.348 (0.114)	0 (0.00)	2 (1.20%)	2 (2.08%)	3.016 (0.221)
Total	97 (91.51)	98 (82.35)	51 (51.52)	49.047 (0.00)	76 (61.79)	106 (80.0)	64 (92.75)	25.522 (0.00)

**Table 3:** Ectoparasites species distribution in goats based on sex age and flock type.

Species of ectoparasites	Sex			Age			Flock type		
	Male (*n* = 112)	Female (*n* = 212)	χ^2^ (*p* value)	Adult (*n* = 168)	Young (*n* = 156)	χ^2^ (*p* value)	Single (*n* = 163)	Mixed (*n* = 161)	χ^2^ (*p* value)
*R*. *evertsi evertsi*	27 (24.11)	81 (38.21)	6.557 (0.010)	83 (49.40)	25 (16.03)	40.556 (0.00)	43 (26.38)	65 (40.37)	7.136 (0.008)
*R*.*B*. *decoloratus*	11 (9.82)	8 (3.77)	4.856 (0.028)	8 (4.76)	11 (7.05)	0.768 (0.381)	3 (1.84)	16 (9.94)	9.621 (0.002)
*A*. *variegatum*	56 (50.00)	102 (48.1)	0.104 (0.747)	94 (55.95)	64 (41.03)	7.214 (0.007)	65 (39.88)	93 (57.76)	10.372 (0.001)
*A*. *gemma*	4 (1.89)	4 (3.57)	0.864 (0.353)	4 (2.38)	4 (2.56)	0.011 (0.915)	5 (3.07)	3 (1.86)	0.488 (0.485)
*L*. *stenopsis*	36 (32.14)	48 (22.64)	3.445 (0.063)	39 (23.21)	45 (28.85)	1.336 (0.248)	39 (23.93)	45 (27.95)	0.683 (0.409)
*C*. *felis*	1 (0.89)	4 (1.89)	0.477 (0.490)	3 (1.79)	2 (1.28)	0.135 (0.713)	5 (3.07)	0 (0.00)	5.016 (0.025)
*C*. *canis*	26 (23.21)	34 (16.04)	2.501 (0.114)	15 (8.93)	45 (28.85)	21.267 (0.00)	33 (20.25)	27 (16.5)	0.648 (0.421)
*S*. *scapie var*. *caprea*	2 (1.38%)	2 (0.71)	0.453 (0.501)	2 (0.96%)	2 (0.92)	0.002 (0.966)	3 (1.33)	1 (0.50)	0.772 (0.379)
Total	84 (75.00)	162 (76.42)	0.080 (0.777)	134 (79.76)	112 (71.79)	2.809 (0.094)	112 (68.71)	134 (83.23)	9.340 (0.002)

**Table 4:** Ectoparasites species distribution in sheep by agroecology and body condition scores.

Species of ectoparasites	Agroecology				Body condition scores			
	Highland (*n* = 21)	Midland (*n* = 11)	Lowland (*n* = 70)	χ^2^ (*p* value)	Good (*n* = 41)	Medium (*n* = 36)	Poor (*n* = 25)	χ^2^ (*p* value)
*R*. *evertsi evertsi*	12 (57.14)	6 (54.55)	40 (57.14)	0.027 (0.987)	22 (53.66)	20 (55.56)	16 (64.00)	0.716 (0.699)
*R*. *B*. *decoloratus*	7 (33.33)	0 (0.00)	0 (0.00)	28.980 (0.001)	2 (5.00)	4 (11.11)	1 (4.00)	71.892 (0.001)
*A*. *variegatum*	17 (80.95)	9 (81.82)	1 (1.43)	71.081 (0.001)	11 (26.83)	12 (33.33)	4 (16.00)	2.282 (0.319)
*A*. *gemma*	0 (0.00)	0 (0.00)	12 (17.39)	6.217 (0.045)	5 (12.20)	1 (2.78)	6 (24.00)	6.414 (0.040)
*C*. *canis*	2 (9.52)	0 (0.00)	5 (7.14)	1.052 (0.591)	5 (4.88)	3 (8.33)	2 (8.00)	0.425 (0.809)
Total	18 (85.71)	11 (100.00)	46 (65.71)	7.759 (0.021)	27 (65.85)	27 (75.00)	21 (84.00)	2.689 (0.261)

**Table 5:** Ectoparasites species distribution in sheep by sex, age, and flock type.

Species of ectoparasites	Sex			Age			Flock type		
	Male (*n* = 34)	Female (*n* = 68)	χ^2^ (*p* value)	Adult (*n* = 41)	Young (*n* = 61)	χ^2^ (*p* value)	Single (*n* = 62)	Mixed (*n* = 40)	χ^2^ (*p* value)
*R*. *evertsi evertsi*	25 (73.53)	33 (48.53)	5.776 (0.016)	25 (60.98)	33 (54.10)	0.473 (0.492)	33 (53.23)	25 (62.50)	0.853 (0.356)
*R*.*B*. *decoloratus*	4 (11.76)	3 (4.41)	1.917 (0.166)	2 (4.88)	5 (8.20)	0.423 (0.516)	2 (3.23)	5 (12.50)	3.272 (0.070)
*A*. *variegatum*	9 (26.47)	18 (26.47)	0.000 (1.000)	13 (31.71)	14 (22.95)	0.965 (0.326)	17 (27.4)	10 (25.00)	0.073 (0.787)
*A*. *gemma*	5 (14.71)	7 (10.29)	0.425 (0.514)	6 (14.63)	6 (9.84)	0.544 (0.461)	5 (8.06)	7 (17.50)	2.085 (0.149)
*C*. *canis*	1 (2.94)	6 (8.82)	1.227 (0.268)	0 (0.00)	7 (11.48)	5.052 (0.025)	4 (6.45)	3 (7.50)	0.042 (0.838)
Total	26 (76.47)	49 (72.06)	0.227 (0.634)	32 (78.05)	43 (70.49)	0.719 (0.396)	44 (70.97)	31 (77.50)	0.533 (0.465)

## Data Availability

All relevant data generated and analyzed during this study are available within the article.
